# Engineering dual-glycan responsive expression systems for tunable production of heterologous proteins in *Bacteroides thetaiotaomicron*

**DOI:** 10.1038/s41598-019-53726-w

**Published:** 2019-11-22

**Authors:** Darryl R. Jones, Marshall B. Smith, Richard McLean, Julie M. Grondin, Carolyn R. Amundsen, G. Douglas Inglis, Brent Selinger, D. Wade Abbott

**Affiliations:** 1 Lethbridge Research and Development Centre, Agriculture and Agri-Food Canada, Lethbridge, Alberta T1J 4B1 Canada; 2 Department of Biological Sciences, University of Lethbridge, Lethbridge, Alberta T1K 3M4 Canada

**Keywords:** Expression systems, Microbial genetics

## Abstract

Genetically engineering intestinal bacteria, such as *Bacteroides thetaiotaomicron* (*B. theta*), holds potential for creating new classes of biological devices, such as diagnostics or therapeutic delivery systems. Here, we have developed a series of *B. theta* strains that produce functional transgenic enzymes in response to dextran and arabinogalactan, two chemically distinct glycans. Expression systems for single glycan induction, and a novel “dual-glycan” expression system, requiring the presence of both dextran and arabinogalactan, have been developed. In addition, we have created two different chromosomal integration systems and one episomal vector system, compatible with engineered recipient strains, to improve the throughput and flexibility of gene cloning, integration, and expression in *B. theta*. To monitor activity, we have demonstrated the functionality of two different transgenic enzymes: NanoLuc, a luciferase, and *Bu*GH16C, an agarase from the human intestinal bacterium, *Bacteroides uniforms* NP1. Together this expression platform provides a new collection of glycan-responsive tools to improve the strength and fidelity of transgene expression in *B. theta* and provides proof-of-concept for engineering more complex multi-glycan expression systems.

## Introduction

The lower digestive tract of mammals contains diverse and abundant bacterial species, collectively referred to as the distal gut microbiota (DGM)^1^. The total collection of genes encoded within the DGM greatly outnumbers the host genome, and encode essential functions, such as digestion of dietary fiber and the synthesis of vitamins and amino acids^2^. In recent years, public and scientific interest in the DGM has intensified as mechanistic relationships between the DGM and host health have begun to be defined^3^. The DGM is required for intestinal and immune system development and function^4^ and can reduce pathogen colonization by competitive exclusion^5^. Imbalances in community structure, termed dysbiosis^6^, have been linked with chronic health conditions such as diabetes^7^, obesity^8^, and inflammatory bowel disease^9^. These issues have been exacerbated by Western diets, which are low in dietary fiber^10^; and the prophylactic and therapeutic use of broad spectrum antibiotics^11^.

*Bacteroides thetaiotamicron* (*B. theta*) is a prominent member of the human DGM. It is detectable in nearly half of healthy Western fecal microbiomes^12^ and is one of the most studied members of Bacteroidetes^13^. Members of this phylum are known for their ability to saccharify a wide variety of chemically complex polysaccharides from diverse biological sources, including terrestrial plants^2^, seaweed^14^, bacteria^15^, fungi^16^, and animals^17^. Glycan metabolism in Bacteroidetes is genetically encoded within polysaccharide utilization loci (PULs)^18^, which are clusters of co-regulated genes typically comprised of TonB-dependent SusC/SusD-like transporter systems, regulatory proteins, and carbohydrate active enzymes (CAZymes) specific for the consumption of discrete substrates. PULs are activated by one of three classes of regulators, including SusR-like, AraC hybrid two-component systems (HTCSs), and extracytoplasmic function (ECF) σ/anti- σ systems^19,20^. Although these regulatory proteins operate through different mechanisms they serve an analogous function within the cell, which is to detect products enzymatically released from a substrate and induce PUL expression.

Augmenting the metabolism of intestinal bacteria, such as *B. theta*, with transgenic enzymes and transporters is a promising approach for establishing and reprogramming established DGM structure. The introduction of enzymes with new functions or that operate at faster rates can release more fermentable sugars^21^, reduce complications associated with slow transit of ingesta^22,23^, and provide a selective advantage in a nutrient competitive ecosystem. Previously, the transfer of a fructan specific SusC/SusD-like transporter endowed the recipient *B. theta* strain with the ability to metabolize a chemically distinct glycan^24^. Recently, *Bacteroides stercoris* and *B. theta* were engineered to utilize porphyran, a structural polysaccharide found in the cell walls of red algae^25^. This transfer provided access to a “privileged” nutrient, and selective feeding on porphyran enabled engraftment of the engineered strain into an established microbiota. Such engineering strategies could usher in a new era of personalized medicine and microbiome engineering.

Despite these advances, genetic elements for tunable and tightly controlled expression of heterologous proteins in bacterial strains adapted for long-term host colonization are still required. *Bacteroides* spp. have unique regulatory and conjugation elements^25–28^, and genetic tools developed for other organisms generally are not tractable in *B. theta*. In this regard, several *Bacteroides*-specific tools have been developed. The pExchange plasmid for homologous recombination has existed since 1991^29^. Using this counter-selectable system, unmarked chromosomal deletions and insertions can be made with single base pair resolution^30,31^. Alternatively, pNBU2, a plasmid containing a mobilizable transposon element^32^ facilitates integration of the entire plasmid indiscriminately into one of two tRNA^SER^ sites^30^. More recently, constitutive promoter elements and ribosome binding sites (RBS) have been screened and ranked to provide a wide-range of expression levels for heterologous proteins^31,33^. Episomal and chromosomal inducible gene expression systems also exist, and they can be regulated by anhydrotetracycline, a synthetic chemical inducer^31^, or the polysaccharide α-mannan^34^. Activation of each of these systems requires the addition of a single inducer for transgene expression.

To increase the fidelity of glycan responsive episomal and chromosomal expression systems in *B. theta* we have altered the natural regulatory mechanisms of PULs by placing them behind dextran (DX)^35^ and arabinogalactan (AG)^20^ responsive promoters. In addition, we have engineered a first in-in-class dual-glycan expression platform that is dependent upon the presence of two chemically distinct glycans that induce a minor diauxic growth phenotype in *B. theta*^36^. This dual-glycan expression system provided improved regulatory control of transgene expression in *B. theta* and enhanced the level of activity of two different reporter enzymes (e.g. a luciferase and agarase) from two different genomic loci and an expression plasmid. Importantly, most of these genetic modifications were not deleterious to bacterial fitness *in vitro*. This study provides unique insights into promoter engineering for improved control of gene expression in *B. theta*, and presents a platform for the future development of other multi-glycan responsive expression systems.

## Results

### Construction of a chromosomal platform for targeted insertion and expression of transgenes

**Figure 1:**
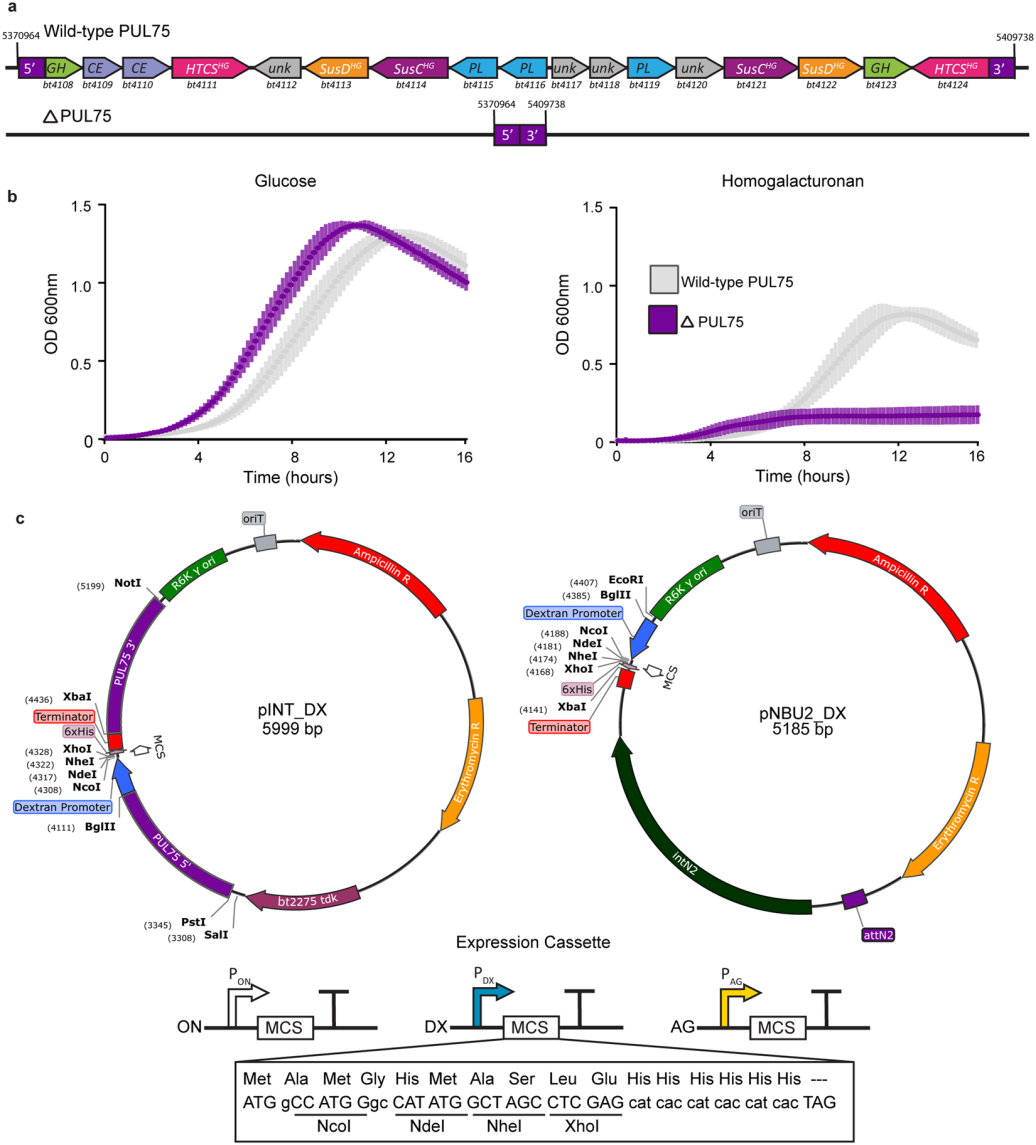
Target genomic locus and integrative expression vectors. **(a)** Schematic of *B. theta* genomic locus PUL75 containing genes required for homogalacturonan (HG) utilization (top). These genes are deleted in the *B. theta* ΔPUL75 strain (bottom). **(b)** Growth profiles of wild-type (WT) *B. theta* (grey) and the ΔPUL75 strain (purple) in MM containing either glucose (GLC) or HG as sole carbon source. Growth was measured by OD_600_ readings over the course of 16 h. Values represent the average of three biological replicates. Variation is represented by standard error of the mean (s.e.m.) **(c)** Plasmid maps of the pINT (left) and pNBU2 (right) vectors. The orientation of the DX, AG, and ON, expression cassettes and the sequence of the MCS are indicated.

In developing a recombinant chromosomal expression system, we sought to recommission the genome space of a characterized PUL. *B. theta* PUL75 (Fig. 1a) is dedicated to the metabolism of homogalacturonan (HG), a pectic glycan comprised of galacturonic acid^37^. Compared to the wild-type strain (WT), *B*. *theta* with a deletion of PUL75 (*B*. *theta*-ΔPUL75; base pairs: 5371714…5408988) was severely restricted in its ability to grow on minimal medium (MM) containing HG as sole carbon source (Fig. 1b;^37^). The vacated genome space in *B*. *theta*-ΔPUL75 was targeted for chromosomal integration using a complementary PUL75-5′ flank and PUL75-3′ flank sequence inserted into the pExchange backbone (Fig. 1c). In addition, a multiple cloning site (MCS) flanked by an upstream RBS, downstream terminator, and inducible or constitutive promoters were placed between the PUL75-5′ and PUL75-3′ sequences. This vector series, referred to as pINTEGRATE (pINT), enables precise and unmarked insertion of expression cassettes into the chromosome of *B. theta*-ΔPUL75. To complement this vector series we developed a pNBU2 plasmid series in parallel that contained an identical MCS and expression elements^30^. The pNBU2 vectors undergo discriminatory single-cross over integration of the entire plasmid into the *B. theta* genome at one of two tRNA^ser^ genes. This system enables faster evaluation of chromosomal transgene function from a second genome locus, but the modified strain is left with residual plasmid DNA and selectable markers in its genome.

### Validation of *B. theta* promoters for regulation of transgenes

**Figure 2:**
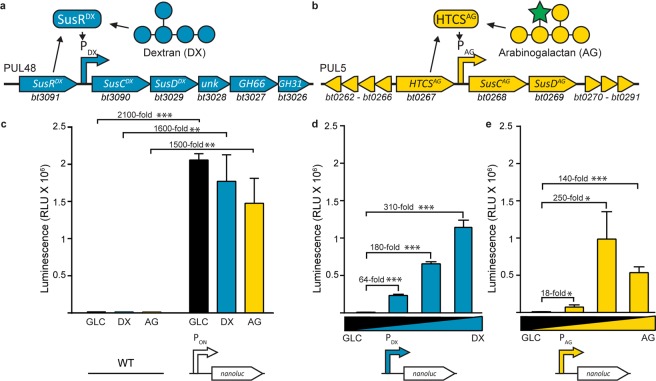
Inducible expression cassettes generate a dose dependent response in NanoLuc activity in *B. theta*. (**a)** Schematic of wild-type regulation by PUL48. P_DX_ is activated by the SusR-like protein SusR^DX^ in the presence of DX. **(b)** Schematic of wild-type AG regulation. P_AG_ from PUL5 is activated by the HTCS protein HTCS^AG^ in response to the presence of AG. Schematic representations of monomeric glucose (GLC; blue circle) the polymer dextran (DX) composed of a chain of GLC; and arabinogalactan (AG), composed of a galactose (GAL, yellow circle) backbone and arabinose side chains (green star). **(c)** NanoLuc activity under constitutive expression by the P_ON_ promoter is measured post induction with GLC (black bar), DX (blue bar), and AG(yellow bar). Luminescence signal for NanoLuc production under the **(d)** DX or **(e)** AG inducible promoters in the presence of increasing concentrations of DX or AG, respectively. Values in (**d–f**) represent the average and s.e.m. for three biological replicates. Asterisks represent levels of significance (*p < 0.05, **p < 0.01, ***p < 0.001).

Three different promoters were selected for insertion into pINT and pNBU2 vectors (Fig. 1c) based upon previous studies. These included a strong constitutive promoter P_ON_ (*bt1311* promoter and the rpiL* RBS^33^), and two inducible promoters: the DX responsive “P_DX_”, which drives expression of the PUL48 *susC*-like gene *bt3090*^35^ (Fig. 2a); and the AG responsive “P_AG_”, which drives expression of the PUL5 *susC*-like gene *bt0268*^33^ (Fig. 2b). DX and AG were selected because they induce some of the strongest glycan responsive expression in *B. theta*, have distinct chemical structures (Fig. 2a,b), are from diverse biological sources (DX is from a bacterial glycan; AG is a plant cell wall glycan), and are induced by distinct regulatory mechanisms; PUL48 uses a SusR-like system (Fig. 2a) and PUL5 uses a HTCS system (Fig. 2b). Despite performing analogous functions in the cell, the SusR-like and HTCS regulators are structurally unrelated^19^. SusR-like proteins are relatively rare, with only five homologs identified in *B. theta*, two of which regulate the PULs involved in the metabolism of chemically distinct glucans^19^. The first SusR protein was discovered in the Sus PUL^38^ and its mechanism remains undefined. HTCSs are the most abundant PUL regulatory proteins in *B. theta*^19^. They are fused integral membrane proteins with a periplasmic exposed carbohydrate binding domain that operate through a “scissor blade-like” mechanism^39^ and a cytoplasmic AraC DNA-binding domain. Following carbohydrate binding, HTCSs undergo a histidine-aspartate phosphorylation cascade and bind DNA as a dimer^36,40,41^.

The ability of each promoter to drive transgene expression was determined using NanoLuc, a luciferase, as a reporter gene^33^. To evaluate proximity effects and differential expression platforms, NanoLuc-pINT and NanoLuc-pNBU2 vectors were conjugated into *B. theta*. Luciferase activity assays established that although genome location resulted in statistical differences in luciferase activity under some conditions, they were small when compared to glycan-induced expression (Fig. S1, *P* < 0.05 in 12/40 comparisons). When grown on glucose (GLC), DX, or AG, the P_ON_ constructs consistently produced luminescence at levels that were three orders of magnitude (*P* < 0.01) above the baseline produced by strains without the NanoLuc gene (Fig. 2c), and P_DX_ and P_AG_ constructs responded to DX or AG in a dose dependent fashion, respectively (Fig. 2d). Intriguingly, P_AG_ displayed higher activity in the presence of GLC and AG than with AG alone (Fig. 2e).

### Engineering tunable expression of glycan-responsive PUL regulator proteins in *B. theta*

Next, we attempted to increase the heterologous production of NanoLuc in *B. theta*, by modifying the induction status of the PUL48 and PUL5 regulatory proteins within the cell. Typically, regulatory proteins, such as HTCS and SusR-like homologs, are constitutively expressed at low levels and are not induced along with CAZymes and transport proteins during glycan metabolism^35^. We reasoned that placing strong constitutive or inducible promoters upstream of the regulator gene in PUL5 and PUL48 may create feedback loops that result in higher levels of expression. Therefore, *B. theta* strains with a series of different promoter architectures were generated (Fig. S2). These included: (1) a constitutive promoter, (2) a positive feedback loop (DX → DX; AG → AG), and (3) alternate forms of hybrid-induction (i.e. DG → AG and AG → DX). (1) The DX constitutive strain was created by placing the P_ON_ *bt1311* promoter^33^ upstream of *bt3091*, the PUL48 SusR-like gene (SusR^DX^), to generate the strain *B. theta*-P_ON_SusR^DX^. The AG constitutive strain was created by placing P_ON_ upstream of *bt0267*, the PUL5 HTCS gene (HTCS^AG^), to generate *B. theta*-P_ON_HTCS^AG^. (2) The positive feedback systems were created by placing P_DX_ upstream of SusR^DX^ to create *B.theta-*P_DX_SusR^DX^; and P_AG_^33^ upstream of HTCS^AG^ to create *B.theta-*P_AG_HTCS^AG^. (3) The hybrid-induction systems were created by inserting P_AG_ in front of SusR_DX_ and P_DX_ in front of HTCS_AG_ to create *B. theta*-P_AG_SusR^DX^, and *B. theta*-P_DX_HTCS^AG^, respectively. In addition, a third hybrid strain was created, *B. theta*-P_AG_SusR^DX^ + P_DX_HTCS^AG^, which contained both hybrid regulatory modifications (Fig. S2).

**Figure 3:**
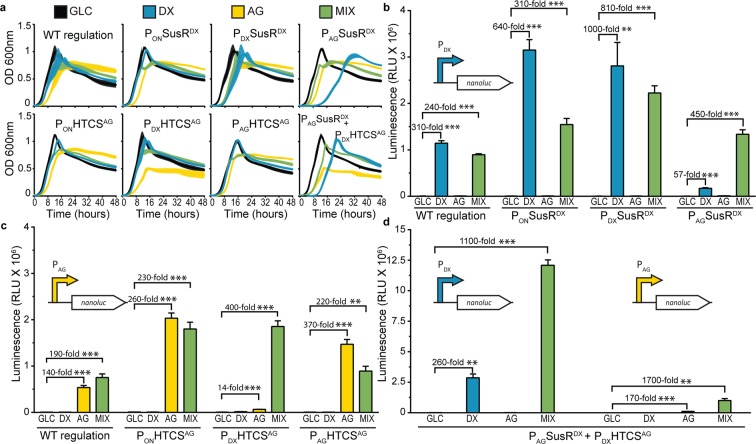
Engineering regulatory elements alters growth profiles, levels of gene activity, and gene induction requirements. (**a)** Growth profiles of WT *B. theta* and engineered regulatory strains on GLC (black), DX (blue), AG (yellow), and a mixture of DX and AG (green). **(b)** Luminescence detected from DX-responsive NanoLuc in strains with engineered *SusR*^*DX*^ when grown on GLC (black), DX (blue), AG (yellow), and the mixture of DX and AG (green). **(c)** Luminescence detected from AG-responsive NanoLuc in strains with engineered *HTCS*^*AG*^ when grown on GLC (black), DX (blue), AG (yellow), and the mixture of DX and AG (green). **(d)** Luminescence detected from both DX- and AG-responsive NanoLuc in a strain with engineered *SusR*^*DX*^ and *HTCS*^*AG*^ when grown on GLC (black), DX (blue), AG (yellow), and the mixture of DX and AG (green). Error bars in (**a**) represent standard deviation (s.d.) and error bars in (**b–d**) represent s.e.m. for three biological replicates. Asterisks represent levels of significance (*p < 0.05, **p < 0.01, ***p < 0.001).

To determine the effect of the modifications to the regulatory proteins of PUL5 and PUL48, the growth profiles of each *B. theta* strain were determined (Fig. 3a). Strains with constitutive regulation (*B. theta*-P_ON_SusR^DX^ and *B. theta*-P_ON_HTCS^AG^) and positive feedback regulation both performed similar to wild-type. Each hybrid strain displayed a growth defect when cultured solely on the glycan sensed by the regulatory protein (DX for P_AG_SusR^DX^, or AG for P_DX_HTCS^AG^, Fig. 3a); however, this defect was mitigated when the strains were cultured on a mixture of DX and AG. This phenotype may be a result of the hybrid strains having a lower basal level of expression when regulated by a glycan-responsive promoter. Whereas on the mixture, the promoters are fully activated and lead to sufficient regulatory protein production to effectively activate their PULs preventing a growth defect.

To examine the effects of the genetic modifications to the regulation of PUL5 and PUL48 on the expression of a transgene, NanoLuc activity was measured in the engineered *B. theta* strains. When placed under SusR^DX^ regulation, the P_ON_ and P_DX_ promoter increased activity approximately three-fold over wild-type in both the pINT (Fig. 3b) and pNBU2 (Fig. S1) platforms (*P* < 0.01). The hybrid strain, *B.theta-*P_AG_SusR^DX^, displayed the lowest NanoLuc signal on DX but similar levels on the DX-AG mixture (*P* < 0.001), which is consistent with what was observed in the growth profiles (Fig. 3a). The AG-responsive systems displayed similar patterns, with a lower increase in NanoLuc activity in the P_ON_ and P_AG_ systems (Fig. 3c, *P* < 0.01).

In contrast to the strains with single modifications, the strain with both hybrid regulator systems (*B. theta*-P_AG_SusR^DX^ + P_DX_HTCS^AG^) displayed noticeable increases (*P* < 0.01) in NanoLuc activity (Fig. 3d). This strain produced the highest luminescence values (*P* < 0.001) for chromosomal NanoLuc in this study when NanoLuc was placed behind P_DX_ and the strain was cultured on mixed sugars. Although it did not reach the same absolute value for reporter activity, NanoLuc positioned behind P_AG_ exhibited the greatest relative difference compared to baseline luminescence on GLC (*P* < 0.01), owing to the high baseline of P_DX_NanoLuc. These results suggest that DX responses are preferential over AG responses in *B. theta* for these systems.

### Augmenting transgene expression using a *B. theta* episomal expression vector

**Figure 4:**
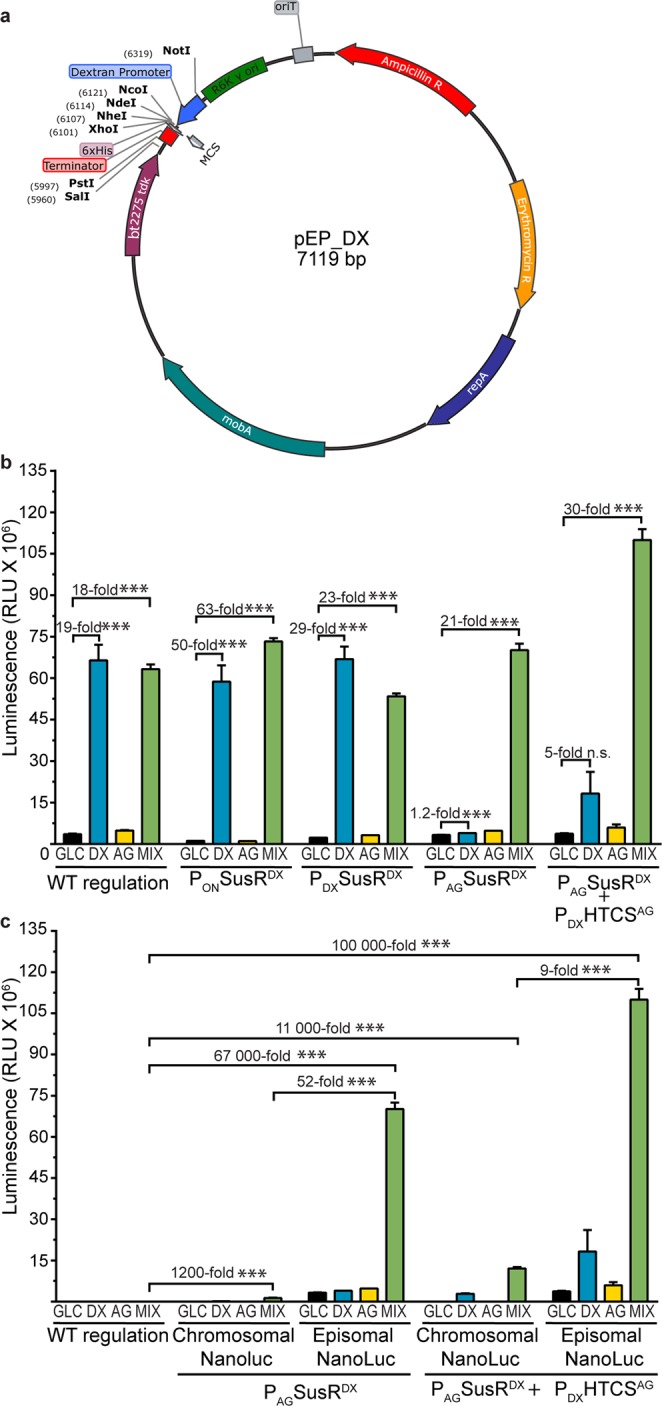
Episomal transgene expression results in higher enzyme activity when compared to chromosomal-based expression. **(a)** Vector map of the extra-chromosomal expression pEP_DX vector. **(b)** Luminescence signal detected from wild-type (WT), *P*_*ON*_*SusR*^*DX*^*, P*_*DX*_*SusR*^*DX*^*, P*_*AG*_*SusR*^*DX*^, and *P*_*DX*_*HTCS*^*AG*^ + *P*_*AG*_*SusR*^*DX*^ strains containing the NanoLuc under the control of the episomal pEP_DX promotor, in the presence of GLC (black bars), DX (blue bars), AG (yellow bars) or the DX and AG mixture (green bars). **(c)** Comparison of luminescence from engineered regulatory strains when NanoLuc is encoded in a plasmid or in the chromosome. Error bars represent s.e.m. for three biological replicates. Asterisks represent levels of significance (*p < 0.05, **p < 0.01, ***p < 0.001).

Plasmid-based expression systems hold several advantages over chromosomal expression systems, including efficient transformation, higher gene copy numbers, and shortened timelines due to lack of requirement for double cross-over selection methods. Several expression vectors compatible with *Bacteroides* spp. have been reported^31,34^. To compare the potency of the DX and AG regulated expression cassettes developed here with previously described vector expression systems, we created the pEpisomalPromoter (pEP) series of plasmids for *B. theta* (Fig. 4a). The pEP series contain the *mobA* and *repA* genes derived from the *Bacteroides* plasmid pBI143 DNA^41^ to allow the vector to persist and replicate independent of the *B. theta* chromosome. Vectors are maintained by erythromycin selection and are equipped with the P_DX_, P_AG_, or P_ON_ expression cassettes from pINT to select glycan responsiveness. As a proof of concept, the NanoLuc gene was cloned into a pEP vector under the control of the DX promotor, and expression was induced by culturing the cells with GLC, DX, AG or a mixture of DX/AG. Plasmid-based expression resulted in higher (*P* < 0.001) luminescence values when compared to chromosomal expression (Fig. 4b). Combining plasmid-based expression with reciprocal modifications to hybrid promoter regulation amplified (*P* < 0.001) effects for selective expression when compared with chromosomal NanoLuc expression (Fig. 4c). The *B.theta-*P_DX_SusR^DX^ and *B.theta-*P_ON_SusR^DX^ strains transformed with a P_DX_-pEP vector had similar levels (*P* < 0.001) of luminescence (60–75 × 10^6^ RLU) on DX and DX-AG mix, suggesting DX levels were sufficient to maximize expression in both systems. In comparison, the *B.theta-*P_AG_SusR^DX^ strain only displayed activation to similar levels when grown on a mixture of DX and AG glycans (*P* < 0.001). This suggests that basal levels of SusR^DX^ when grown on pure DX was not enough to drive plasmid expression; however, this effect was overcome by adding AG to the medium (Fig. 4b). An approximately two-fold amplification (*P* < 0.001) of NanoLuc activity over the other strains was observed in the *B. theta*-P_AG_SusR^DX^ + P_DX_HTCS^AG^ strain when grown on the AG-DX mix. This suggests that inducing both regulators results in higher levels of expression of heterologous genes using the pEP-plasmid system. *B. theta*-P_AG_SusR^DX^ + P_DX_HTCS^AG^ transformed with P_DX_-pEP had the highest levels of reporter activity in this study. Use of the plasmid with this strain culminated in an order of magnitude increase over the chromosomal system and a six-order of magnitude increase over the background luminescence of *B. theta* WT (Fig. 4c).

### Heterologous production of an agarase in modified *B. theta* strains

**Figure 5:**
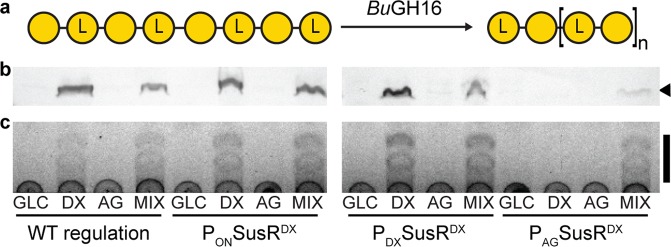
The heterologous *Bu*GH16 agarase is enzymatically functional in engineered *B. theta* strains. (**a)** Schematic model of *Bu*GH16 endo-agarase activity agarose to generate neoagarooligosaccharides (NAOS). NAOS is a repeating disaccharide of l-3,6-anhydro-galactose (L-3,6) and d-galactose, with L-3,6 at the non-reducing end. **(b)** pINT integrated hexa-histidine tagged *Bu*GH16 (black arrow) detected by Western blot from induced *SusR*^*DX*^ strains. **(c)** Thin-layer chromatography of culture supernatant from induced engineered strains incubated with agarose shows the presence of NAOS (black bar) generated from agarose by active *Bu*GH16.

To determine whether the dual-glycan expression systems developed here can be used to produce a heterologous carbohydrate active enzyme, we used the pINT vector to integrate a family 16 glycoside hydrolase from *B. uniformis* NP1 (*Bu*GH16) into the *B.theta-*SusR^DX^ strain series. *Bu*GH16 is an endo-β-agarase that cleaves agarose to produce neoagarotetraose as a terminal product^42^ (Fig. 5a). Wild-type *B. theta* does not possess this catalytic activity. *Bu*GH16, fused to a N-terminal outer membrane anchoring tag, was cloned into the pINT- P_DX_ vector and integrated into the *B. theta*-SusR^DX^ strain. The outer membrane tag enabled whole-cell agarolysis assays to be performed as the enzyme cargo is displayed on the outer surface. For the *B. theta*-P_ON_SusR^DX^ and *B. theta*-P_DX_SusR^DX^ strains, transgenic *Bu*GH16 protein activity was detected and agarose activity was observed in all conditions containing DX (Fig. 5b). Production of *Bu*GH16 in the P_AG_SusR^DX^ multi-glycan induction strain only displayed *Bu*GH16 activity when the cultures were treated with both DX and AG. This pattern is similar to the NanoLuc activity in the dual-glycan pINT (Fig. 3b) and pEP vector (Fig. 4b) systems. Taken together, these results suggest that engineering HTCS and SusR-like regulator proteins to respond to mixtures of chemically defined glycan inducers in chromosomal and plasmid-based systems can improve the regulatory fidelity of heterologous enzyme production in *B. theta*.

## Discussion

Engineering intestinal bacteria to express heterologous proteins is a promising approach to improve host health. Previous studies with *B. theta* have developed expression cassettes that are constitutive^33^, or respond to common glycans^33,34^ or synthetic chemicals^31^. We have expanded upon this palette of genetic parts to develop the pINT, pNBU2, and pEP-based gene expression systems. pINT constructs specifically integrate its genetic cargo into the vacated genome space of *B. theta*-ΔPUL75. This genome space was selected to prevent large increases to the genome size and to repurpose a decommissioned locus known to be involved in metabolism of a glycan. Each expression platform has been designed for convenient subcloning of target genes, tailoring of promotor selectivity, and if desired, altering the targeted integration site within the genome as each DNA segment is flanked by directional restriction sites (Fig. 1c). Three different validated promoters were inserted into these expression cassettes, including P_ON_ (*bt1311*), a strong constitutive promoter; and P_DX_ (*bt3090*) and P_AG_ (*bt0267*) responsive promoters, two selective glycan responsive elements, P_AG_ and P_DX,_ that respond to chemically distinct glycans^35^ (Figs. 2a,b, S2). Constitutive expression of NanoLuc driven by P_ON_ was 1,500 to 2,000-fold greater than baseline auto-luminescence, on three different carbon sources (Fig. 2c). These results are consistent with previous findings^33^, and underpin that this constitutive promoter operates independent of glycan-specific regulatory networks (Fig. 2c). Surprisingly, DX and AG induction of NanoLuc resulted in different activity profiles. DX displays a conventional dose-dependent relationship when mixed with GLC (Fig. 2d); whereas, the AG-responsive promoter displayed optimal NanoLuc activity at 50:50 (GLC:AG), and there was an inhibitory effect observed at when treated solely with AG (Fig. 2e). The AG-responsive promoter used in this study is from PUL5 and was chosen due to previously determined high levels of expression^20^ and proven activity^33^; however, PUL5 is not the sole AG responsive PUL in the *B. theta* genome as PUL65 has also been shown to be involved in AG utilization^20^. When the PUL65 HTCS is deleted and the PUL5 HTCS is left intact, the mutant strain grows to a higher density (150%) on AG than the WT *in vitro*^20^. This indicates that PUL65 may have a repressive effect on PUL5 that is alleviated by deleting the PUL65 HTCS, a hypothesis that could explain the repression on nanoLuc observed in this study. These relationships may be exploited to tailor expression profiles using the AG-responsive system.

To explore the potential of augmenting glycan responsive expression levels and providing higher fidelity in controlling regulation, the promoter elements of *SusR*^*DX*^ and *HTCS*^*AG*^ were engineered to be constitutively expressed under control of P_ON_, and in response to DX and AG (Fig. 3). For both *SusR*^*DX*^ and *HTCS*^*AG*^, constitutive expression and positive feedback had the largest effect, boosting NanoLuc activity three-fold and two-fold above WT for DX (Fig. 3b) and AG (Fig. 3c), respectively. We believe this may be due to increases in the cellular pool of regulatory protein available to sense sugar and upregulate target genes. Supporting this, it has previously been observed that increasing the copy number of the canonical *sus* regulatory gene by incorporating it into a plasmid leads to increases in reporter gene activity^43^. In the case of hybrid-regulation (*P*_*AG*_*SusR*^*DX*^ and *P*_*DX*_*HTCS*^*AG*^), both DX and AG were required for maximal expression. These results suggest that the regulators are not induced on the single sugars, and when both DX and AG are supplied, the promoters become activated and lead to higher levels of target gene expression. This hybrid effect was more pronounced when both SusR^DX^ and HTCS^AG^ were introduced into the same strain (*B. theta*-P_AG_SusR^DX^ + P_DX_HTCS^AG^) and NanoLuc was induced by DX (Fig. 3d). A similar expression profile was not observed with AG induction, however, suggesting that the expression of AG-associated genes may be deprioritized during DX metabolism and that these hybrid systems are glycan specific. Previously, the impact of multiple glycans pairs, including DX and AG, on the growth phenotype of *B. theta* and HTCS phosphorylation was reported^36^. Although the diauxic effect varied for individual glycan pairs, a subtle diauxie for DX and AG was observed, supporting that DX is prioritized over AG metabolism^35^. However, because this growth effect was modest and the growth phenotype on a DX-AG mixture reported here appears to be a blending of the two signal glycan phenotypes rather than a clear diauxic prioritization (Fig. 1a), it suggestions that both DX and AG are digested simultaneously. In this regard, other combinatorial or prioritized glycan metabolic cascades^35,36^ could be exploited to engineer diverse spectrums of dual-glycan response systems, thereby providing further plasticity in tuning the expression of transgenic enzymes.

In order to determine if the dual-glycan effects are conserved using expression vectors, we built the pEP vector system, cloned NanoLuc behind P_DX_, and transformed the SusR^DX^ strain (Fig. 4a). *B. theta*-P_AG_SusR^DX^ produced an aproximately 20-fold increase on the mixed glycans compared to expression on pure glycans (Fig. 4b). Encouragingly, this effect was further amplified in the *B. theta*-P_AG_SusR^DX^ + P_DX_HTCS^AG^ strain (Fig. 3d). The higher luminescence activity observed using pEP-expression is attributed to the potential for higher copy numbers of the expression cassette within the cell. These vectors replicate within the cell independently of the genome as pBI143, the natural plasmid containing the mobilization and replication genes that were inserted into pEP, has been estimated to be maintained at a copy number of 20 per cell^41^. Additionally, it may result due to proximal effects of regulation. The SusR-like regulatory proteins are integral membrane proteins, which may require proximity to its target genes to maximally activate their expression. Episomal expression vectors may overcome this obstacle by diffusing to the cytoplasmic DNA-binding domain of transmembrane regulatory proteins.

To demonstrate that the dual-glycan expression system is compatible for a second transgenic enzyme, we replaced NanoLuc with *Bu*GH16, an agarase from *B. uniformis* NP1. *Bu*GH16 cleaves the β-d-galactose-(1,4)-3,6-anhydro-l-galactose bond in agarose^42^ (Fig. 5a). We compared *Bu*GH16 activity with the P_DX_ promoter in *B. theta* strains with modified SusR^DX^*. Bu*GH16 production (Fig. 5b) and agarase activity (Fig. 5c) was observed when induced by DX, and DX and AG mixtures in the control, P_ON_SusR^DX^ and P_DX_ SusR^DX^ strains. Consistent with NanoLuc, dual-glycan regulation was only observed in the P_AG_-SusR^DX^ system when *Bu*GH16 was induced by a mixture of DX and AG. The observation that the hybrid-systems require two chemically distinct glycans for expression represents a new engineering solution for regulating transgene expression in *B. theta*. Other expression systems requiring two unique molecules for induction have been described, including the single chain tetracycline repressor in *E. coli* that requires both tetracycline and 4-dedimethylamino-anhydrotetracycline^44^, or the classic *lac* operon that requires both lactose and cyclic adenosine monophosphate bound catabolite activator protein for full expression^45,46^. The use of a dual-glycan expression system reported here, however, has the advantage of being regulated by common dietary glycans.

## Conclusion

Gene expression systems with unique induction requirements for transgene expression have been created in *B. theta* by modifying the promoter elements of regulatory genes from two distinct PULs. These systems have been shown to increase expression of two different transgenes spanning a dynamic range of 2,000-fold^34^ to 100,000-fold^31^, consistent with other studies. Most significantly, our discovery that engineering regulatory gene promoters to be activated by chemically distant glycans (i.e. DX and AG), resulted in a dual-glycan expression system that is dependent on both glycans to maximize gene expression. This property was reproduced using integration plasmids that target two different genomic loci, and from pEP, an in-house designed episomal expression system. The results from this study sets the stage for engineering more complex glycan-directed regulatory engineering and rationally designed bacterial tools that respond to dietary glycans.

## Methods

### Vector construction

The recipient strain, *B. theta*-ΔPUL75, was previously created by counter selectable homologous recombination^37^. PUL75 (genome base pairs 5371714…5408988) was targeted for chromosomal deletion inserting complementary flank sequences inserted into the pExchange backbone. To generate the complementary pINTEGRATE vectors (pINT_DX) a 750 bp region upstream of PUL75 (PUL75 5′) and the promoter and initiator codon of *bt3090* were amplified from *B. theta* genomic DNA and assembled by overlap PCR to contain a BglII cut site between the two fragments (to facilitate future promoter variants of pINT) with a PstI cut site on the 5′ end of the insert and an NcoI site on the 3′ end. Concurrently, the terminator from pNBU2^31^ was amplified with a 5′ forward primer containing an MCS with 6xHis tag and a 3′ reverse XbaI primer. After digest with PstI/NcoI and NcoI/XbaI respectively, these inserts were ligated into pExchange-tdk (pEX-tdk)^31^ that were restricted with PstI and XbaI, and transformed into chemically competent S17-1λpir *E. coli* cells. The amplified construct was then digested with XbaI and ligated to a 750 bp PCR fragment corresponding to the downstream region of PUL75 (PUL75 3′) with an inserted XbaI site on the 5′ end and a NheI (XbaI isocaudomer) site on the 3′ end. The construct was confirmed by restriction digest and Sanger sequencing. pINT_AG and pINT_ON were created by replacing the BglII to NcoI fragment in pINT_DX with the promoter and initiator codon from *bt0268* genomic DNA and *bt1311* and the rpiL* RBS from pAT593^33^, respectively.

The single-step integrative vector pNBU2_DX was generated by amplifying the Promoter-MCS fragment from pINT_DX and ligating it into a variant of pNBU2 amplified to create BglII and XbaI cut sites between the R6K origin of replication and the terminator of pNBU2^33^. The promoter and MCS from pINT_AG and pINT_ON were amplified with BglII and XbaI, respectively, and cloned into the reciprocal sites of pNBU2_DX to create pNBU2_AG and pNBU2_ON.

To create the episomal expression constructs pEP_DX, pEP_AG, and pEP_ON, the promoter to terminator fragments from pINT_DX, pINT_AG, and pINT_ON were amplified with NotI and PstI restriction enzyme sites, respectively, and cloned into a variant of pEx-tdk containing the *repA* and *mobA* genes (Genbank AAB39963.1 and AAB39964.1, respectively), which allow the plasmid to exist in *B.theta* extra-chromosomally.

The reporter genes NanoLuc^47^ and *Bu*GH16 (*np1_8*^42^) were fused to respective promoters by overlapping PCR and cloned into the BglII/XhoI or NotI/XhoI sites of the respective vectors to create reporter constructs. In order to target *Bu*GH16 to the cell surface and to avoid interference with the translocation of endogenous proteins, the native N-terminal signal peptide of *Bu*GH16 (nucleotides 1–67) was replaced with that of the putative polysaccharide lyase family 6 (PL6) (*bt4116*; bases 1–63) from PUL75, predicted to be expressed on the surface of *B. theta*^48^.

### Regulatory gene promoter engineering

pEx-tdk was used to insert one of three promoters in front of the start codon of *bt3091* (*SusR*^*DX*^) or *bt0267* (*HTCS*^*AG*^). The three promoters were P_bt1311_ (promoter from pAT593^33^), P_bt3090_ (the contiguous sequence between the stop codon of *bt3091* and the start codon *bt3090*), or P_bt0268_ (promoter from pMM660^33^) and were denoted as P_ON_, P_DX_, and P_AG_ respectively. A terminator sequence was appended to the 5′ end of each promoter to reduce context dependence. The insertions were targeted by cloning the 750 bp flanks on either side of the desired insertion site into the pEx-tdk vector.

### Bacterial conjugations

Donor cultures of *E. coli* strain S17-1λpir were grown in 5 mL lysogeny broth (LB)^49^ with 100 μg/mL ampicillin. Recipient strains of *B. theta* were routinely grown in 5 mL Tryptone Yeast Extract Glucose (TYG)^50^ at 37 °C in an anaerobic atmosphere (85% N_2,_ 10% CO_2_, 5% H_2_). Donor and recipient cultures were pelleted by centrifugation and resuspended together in 1 mL of TYG and plated on Supplemented Brain Heart Infusion (BHIS)^51^ agar. To allow cell mating to occur, cultures were grown agar side down at 37 °C overnight under aerobic conditions. During cell mating, the vector is transferred from the donor *E. coli* and integrated into the chromosome of recipient *B. theta* through homologous recombination.

After 16–24 h, the resulting biomass was scraped from the plate, suspended in TYG broth and serially diluted. Cell suspensions were plated on BHI agar with 200 μg/mL gentamycin to select against *E. coli*, and 25 μg/mL erythromycin to select against *B. theta* that had not received vector. Pure cultures were prepared from arbitrarily-selected resistant colonies.

pNBU2-based conjugations were screened by colony PCR to confirm vector integration. For the double crossover conjugations using pEx-tdk and pINT, eight resistant colonies were arbitrarily chosen and grown in TYG broth. At this stage, a second recombination event may occur excising the vector backbone and selection markers from the chromosome and retaining the sequence of interest. Each cell culture was plated on BHI agar with 200 μg/mL 5-fluorodeoxyuridine (FUDR) to select against cells that retained the plasmid in their chromosome. Pure cultures were screened by colony PCR. Positive clones underwent genome extraction for sequence confirmation.

### Growth curves

Strains of *B. theta* were inoculated from a glycerol stock into 5 mL TYG medium and grown for 16–24 h in an anaerobic atmosphere. TYG cultures were diluted 1 in 50 into prewarmed anaerobic 2 × *Bacteroides* Minimal Medium (MM)^52^. 100 μL of diluted cells were plated in a transparent 96 well plate containing 100 μL of prewarmed anaerobic 1% (w/v) carbohydrate solution to achieve final concentrations of 1X MM and 0.5% (w/v) sugar. Carbohydrates examined were 0.5% (w/v) glucose (GLC), 0.5% homogalacturonan (HG), 0.5% (w/v) dextran (DX), 0.5% (w/v) arabinogalactan (AG), or a mixture of 0.25% (w/v) DX combined with 0.25% (w/v) AG (mix). Plates were sealed with clear, gas permeable membranes, and turbidity was measured in a BioTek Synergy HT plate reader programmed to read absorbance at 600 nm every 10 m for 48 h. Technical triplicates, each being the average of three observations, were tested for each condition. Negative controls consisted of wells containing 100 μL medium and 100 μL 18 MΩcm^−1^ ultrapure H_2_O. The average of the triplicates normalized for media background was plotted with the standard error of the mean.

### NanoLuc assays

Dense TYG pre-cultures were created as described above for growth curves. TYG cultures were diluted 100-fold into prewarmed MM with 0.5% carbohydrate solution in borosilicate glass tubes. After 24 h incubation at 37 °C under anaerobic conditions, cells were pelleted by centrifugation and the supernatant was discarded. Cells were lysed by resuspending in 1/10^th^ culture volume of BugBuster® (Millipore Sigma) and incubating at room temperature for 10 min. Phosphate buffered saline (PBS) was used to dilute lysate 20 to 200-fold to ensure the signal was within the dynamic range of the luminescence detector. 30 μL samples were added to an opaque 96 well microplate and combined with 30 μL of freshly prepared NanoGlo reagent (Promega). Luminescence was measured using a Synergy HT Multi-detection plate reader. Technical replicates, each being the average of three observations, were tested for each condition. The luminescence of the negative controls (30 μL PBS + 30 μL NanoGlo) was used to normal signal for each replicate. The detected value corrected for the dilution factor and normalized to the density of the original culture as shown in Eq. (1).

1 $$\frac{(({\rm{luminescent}}\,{\rm{reading}}-{\rm{negative}}\,{\rm{control}})\times {\rm{dilution}}\,{\rm{factor}})}{{{\rm{OD}}}_{600}\,{\rm{of}}\,{\rm{culture}}}$$

The technical triplicates were then averaged and plotted with the standard error of the mean.

### *Bu*GH16 Agarolysis assays

The *B.theta-*SusR^DX^-*bugh16* strain was culture as described above for the NanoLuc assays; however, cells where not lysed. Whole cells were washed and resuspended in 2X MM before being incubated with an equal volume of 0.8% (w/v) agarose under aerobic conditions at 37 °C for 24 h. The liquid phase was clarified by centrifugation before heat killing at 100 °C for 10 min. Nine μL of the resulting solution was spotted onto a silica thin layer chromatography (TLC) plate. The TLC plate was run in a 2:1:1 (v/v) solution of butanol: acetic acid: 18 MΩcm^−1^ ultrapure H_2_O. After drying, the plate was stained with one part 0.2% (w/v ethanol) dihydroxynaphthalene to two parts 3.75:1 ethanol: sulfuric acid solution, and developed by heating at 100 °C for 5 min.

### Western blotting

*B.theta-*SusR^DX^-*bugh16* cells were lysed in 1:45 of the culture volume BugBuster (Millipore). Cell lysate was mixed 1:1 with SDS loading buffer (10 μL of 2 M glycerol, 1.28 M β-mercaptoethanol, 125 mM Tris pH 6.8, 140 mM SDS, 60 μM bromophenol blue) and denatured at 95 °C for 5 min. Samples were run on 15% sodium dodecyl sulfate polyacrylamide gels at 200 V for 1 h in a Novex Mini Cell (Invitrogen). Protein was transferred from gels to a Sequi-Blot polyvinylidene fluoride membrane (BioRad) in an X Cell II Blot Module (Invitrogen) at 30 V for 16 h. Membranes were incubated with blocking buffer (5% (w/v) skim milk powder in a solution of 50 mM tris, 150 mM NaCl and 0.1% (v/v) tween 20 (TBS-T)) for 2 h followed by incubation with a 1:2500 dilution of rabbit anti-6-his IgG conjugated to horse radish peroxidase (Bethyl) in blocking buffer for 6 h. Membranes were washed three times by rinsing in 18 MΩcm^−1^ ultrapure H_2_O and rocking in TBS-T for 5 min. An Opti-4CN (BioRad) substrate kit was used to develop the membranes.

### Statistical analysis

To examine whether or not changes in NanoLuc activity between strains and conditions were significant, data were analyzed using GraphPad Prism 7.00. T-tests were used to make specific comparisons between conditions. Each condition had three biological replicates and tests were performed without assuming a consistent standard deviation. *P* values of 0.05, 0.01, and 0.001 were used to define levels of significance.

## Supplementary information

Supplementary Information

## Data Availability

Activity assay data is available upon request.
