# Supplementary material for: Engineering dual-glycan responsive expression systems for tunable production of heterologous proteins in Bacteroides thetaiotaomicron
Source: Sci Rep. 2019 Nov 22;9:17400. doi: 10.1038/s41598-019-53726-w (PMC6874557; doi:10.1038/s41598-019-53726-w)
Supplement: Supplementary file 1 — Supplementary Information [file 41598_2019_53726_MOESM1_ESM.pdf]

Engineering dual-glycan responsive expression systems for tunable production of heterologous proteins in *Bacteroides thetaiotaomicron*

Darryl R. Jones[1¶], Marshall B. Smith[1,2¶], Richard McLean[1], Julie M. Grondin[1], Carolyn R. Amundsen[1], G. Douglas Inglis[1,2], L. Brent Selinger[2], and D. Wade Abbott[1,2*]

[1]Lethbridge Research and Development Centre, Agriculture and Agri-Food Canada, Lethbridge, Alberta, Canada, T1J 4B1

[2]Department of Biological Sciences, University of Lethbridge, Lethbridge, Alberta, Canada, T1K 3M4

Figure S1: Comparison of NanoLuc activity from two different genomic loci. pINT integrates NanoLuc into the vacant PUL75 locus. pNBU2 integrates NanoLuc, and the entire pNBU2 plasmid, into a $tRNA^{ser}$ locus. To demonstrate that genomic locus did not affect transgene expression, each recombinant pINT strain was compared to its pNBU2 counterpart. **(a)** NanoLuc activity under control of the P_{ON} promoter when induced by GLC (black), DX (blue), or AG (yellow). **(b)** NanoLuc activity under control of the P_{DX} promoter on varying concentrations of GLC and DX. **(c)** NanoLuc activity under control of the P_{AG} promoter on varying concentrations of GLC and AG. **(d)** NanoLuc activity under control of the P_{DX} promoter in modified $SusR^{DX}$ strains on GLC (black), DX (blue), or AG (yellow) or mixed DX and AG (green). **(e)** NanoLuc activity under control of the P_{AG} promoter in modified $HTCS^{AG}$ strains on GLC (black), DX (blue), or AG (yellow) or mixed DX and AG (green). **(f)** NanoLuc activity under control of either P_{DX} or P_{AG} in strains with modified $SusR^{DX}$ and $HTCS^{AG}$ on GLC (black), DX (blue), or AG (yellow) or mixed DX and AG (green). Significant differences are indicated with brackets. Lack of brackets indicates no significant difference. 32/48 (67%) of comparisons between the two loci do not result in statistically significant difference. Asterisks represent level of significance (*= $p < 0.05$, **= $p < 0.01$, ***= $p < 0.001$).

Figure S2: Schematic regulatory maps of strains engineered in this study. Promoters (colored arrows) are placed upstream of open reading frames (pointed rectangles) on the genome. Blue arrow = dextran responsive (bt3091), yellow arrow = arabinogalactan responsive (bt0268). Regulatory proteins (rounded boxes) are induced by carbohydrates and effect downstream expression of reporter proteins.

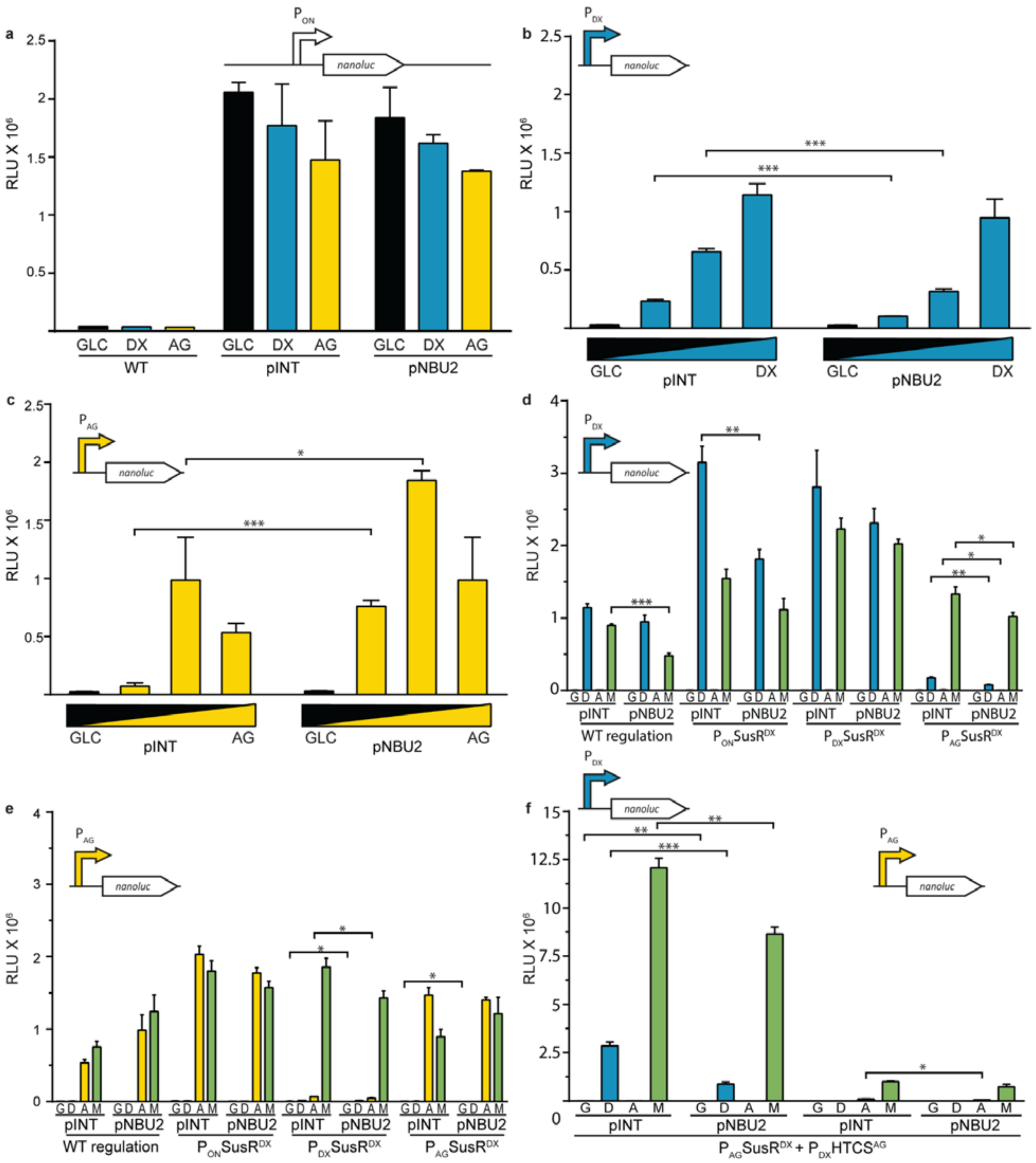

a
P_ON
nanoluc
RLU X 10^6
GLC DX AG GLC DX AG GLC DX AG
WT
pINT
pNBU2
b
P_DX
nanoluc
***
***
GLC pINT DX GLC pNBU2 DX
c
P_AG
nanoluc
*
***
GLC pINT AG GLC pNBU2 AG
d
P_DX
nanoluc
WT regulation
P_ON SusR^DX
P_DX SusR^DX
P_AG SusR^DX
e
P_AG
nanoluc
f
P_AG SusR^DX + P_DX HTCS^AG

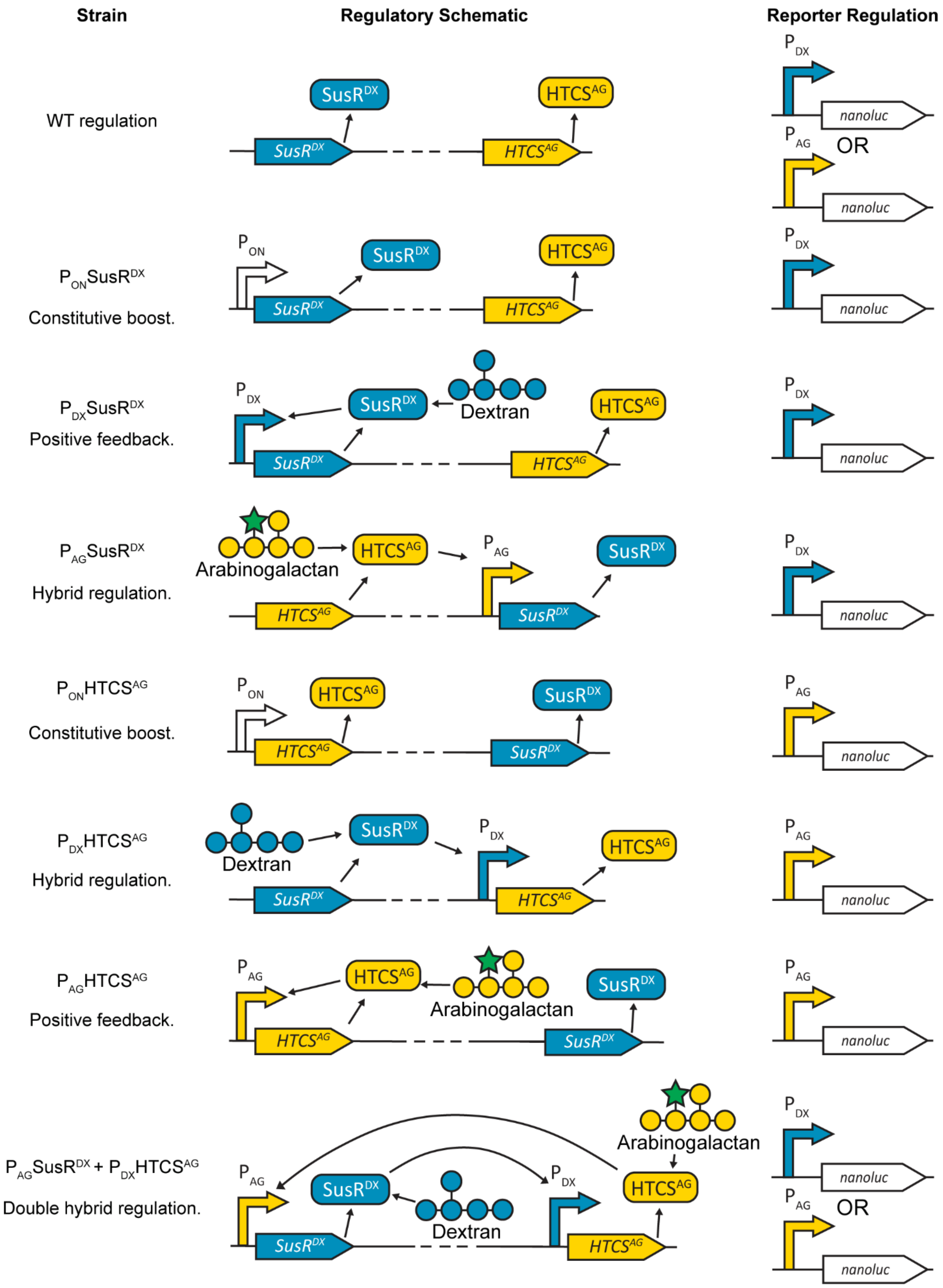
Strain
Regulatory Schematic
Reporter Regulation
WT regulation
$P_{ON}SusR^{DX}$
Constitutive boost.
$P_{DX}SusR^{DX}$
Positive feedback.
$P_{AG}SusR^{DX}$
Hybrid regulation.
$P_{ON}HTCS^{AG}$
Constitutive boost.
$P_{DX}HTCS^{AG}$
Hybrid regulation.
$P_{AG}HTCS^{AG}$
Positive feedback.
$P_{AG}SusR^{DX} + P_{DX}HTCS^{AG}$
Double hybrid regulation.
$SusR^{DX}$
$HTCS^{AG}$
P_{ON}
P_{DX}
P_{AG}
Dextran
Arabinogalactan
nanoluc
OR
